# Redescription and Geographical Distribution of the Endangered Fish *Ossubtus xinguense* Jégu 1992 (Characiformes, Serrasalmidae) with Comments on Conservation of the Rheophilic Fauna of the Xingu River

**DOI:** 10.1371/journal.pone.0161398

**Published:** 2016-09-23

**Authors:** Marcelo C. Andrade, Leandro M. Sousa, Rafaela P. Ota, Michel Jégu, Tommaso Giarrizzo

**Affiliations:** 1 Universidade Federal do Pará, Cidade Universitária Professor José Silveira Netto, Instituto de Ciências Biológicas, Programa de Pós-Graduação em Ecologia Aquática e Pesca, Laboratório de Biologia Pesqueira e Manejo dos Recursos Aquáticos, Belém, PA, Brazil; 2 Universidade Federal do Pará, Campus Universitário de Altamira, Faculdade de Ciências Biológicas, Laboratório de Ictiologia, Altamira, PA, Brazil; 3 Instituto Nacional de Pesquisas da Amazônia, Programa de Pós-Graduação em Biologia de Água Doce e Pesca Interior, Manaus, AM, Brazil; 4 Institut de Recherche Pour le Développement, Biologie des Organismes et Ecosystèmes Aquatiques, Laboratoire d’Icthyologie, Muséum national d’Histoire naturelle, Paris, France; University of Oxford, UNITED KINGDOM

## Abstract

The monotypic species *Ossubtus xinguense* was originally described based on scarce material putatively divided into juveniles and adults. *Ossubtus xinguense* has a restricted distribution and was previously known only from a few rapids downstream of the city of Altamira, in the Volta Grande stretch of the Middle Xingu River. Until recently, the species was rare in museums because its habitat (large rapids) is difficult to sample. Large-scale collecting efforts targeting rapids throughout the Xingu River basin have yielded an abundance of new material. Based on an analysis of the type series and freshly preserved specimens, we redescribe *O*. *xinguense* and provide detailed osteological descriptions along with comments about its relationships within Serrasalmidae. Furthermore, we expand the geographical distribution of the species and discuss its conservation status.

## Introduction

The monotypic genus *Ossubtus* was established by Jégu [1] to include new species *O*. *xinguense*. The original description was based on 15 specimens from rapids in the Xingu River near Altamira city. At that time, the species was thought to be rare in nature and restricted to the vicinity of the type locality [2]. *Ossubtus xinguense* is endemic to the Xingu basin and inhabits rapids with rock outcrops covered by macrophytes of the family Podostemaceae [1], [2]. Those habitats are severely threatened by the recent completion of major construction on the Belo Monte Dam complex on the Xingu River. The poorly known *O*. *xinguense* is easily diagnosed from all other serrasalmids by having mouth subinferior to inferior (versus mouth terminal or upturned). The profile of the head and snout in *O*. *xinguense* resembles that of the rodent capybara, inspiring its Brazilian common name, ‘pacu-capivara’.

**Fig 1:**
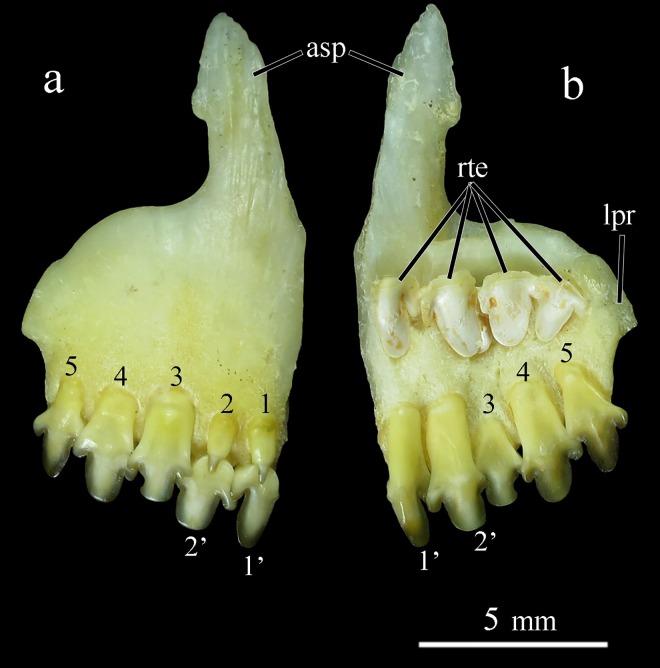
Premaxilla of *Ossubtus xinguense*, GEA 1999, female, 181.7 mm SL, (a) external view, (b) internal view; asp Ascending Process of Premaxilla, lpr Lateral Process of Premaxilla, rte Replacement Teeth, 1–5 Labial premaxillary teeth, 1’-2’ Lingual premaxillary teeth.

**Fig 2:**
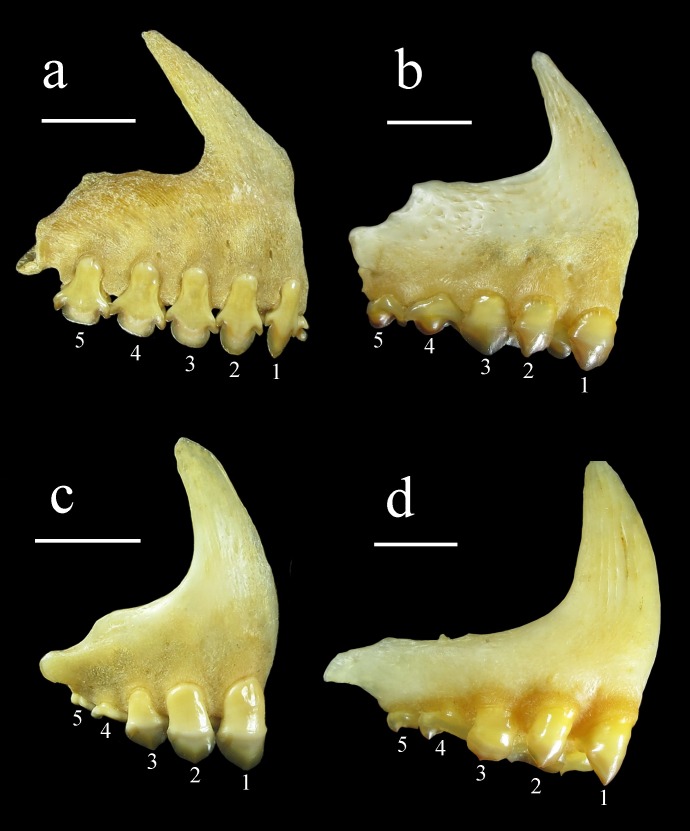
Premaxilla of herbivorous Serrasalmidae in external view, respectively: (a) *Mylesinus paraschomburgkii*, GEA 1185, 62 mm HL; (b) *Tometes* sp. Tocantins, GEA 1945, 280 mm SL; (c) *Myleus setiger*, GEA 1972, 45 mm HL; (d) *Myloplus rubripinnis*, GEA 2238, 58 mm HL. 1–5 Labial premaxillary teeth; Scale bars = 5 mm.

*Ossubtus xinguense* is related to the herbivorous Serrasalmidae named ‘pacus’ [3], and hypothetically nested within a monophyletic clade comprised exclusively of pacus from rapids and waterfalls, the so-called ‘*Myleus*’ clade sensu Ortí *et al*. [4]. That clade also includes *Myleus*, *Myloplus*, *Tometes* and *Mylesinus*, however, the monophyly of those genera is not supported [4]. Jégu [5], in his unpublished PhD dissertation on serrasalmid relationships, proposed a monophyletic clade formed by *Myleus-Tometes-Mylesinus-Ossubtus* based on 12 synapomorphies highlighting the morphology of the jaws and olfactory fossa: dorsal and lateral processes of premaxilla slender and thin; premaxillary teeth in two rows contacting each other; incisiform teeth specialized for cutting aquatic macrophytes; and olfactory fossa wide (versus the sister clade *Myloplus* with premaxillary teeth in two rows separated from each other; molariform teeth specialized for crushing seeds; and a narrow olfactory fossa). The clade sensu Jégu [5] is further divided into *Myleus-Tometes* group with robust incisiform teeth strongly attached to jaws versus *Mylesinus-Ossubtus* group with fragile and weakly attached incisiform teeth [6]. Furthermore, in the original description, Jégu [1] noticed that the first two labial premaxillary teeth of *O*. *xinguense* are weak and canine in shape, thereby differing from the remaining premaxillary teeth (Fig 1A). That condition was previously observed only in juvenile specimens of *Mylesinus* and *Tometes* up to 45 mm standard length [7], [8], [9]. In the other pacu genera, the first two labial teeth resemble the remaining premaxillary teeth (Fig 2A–2D). Jégu [5] treated the anterior labial caniniform teeth in *O*. *xinguense* as an autapomorphy. However, while examining these two first teeth in *O*. *xinguense* the authors noticed a pattern different from original description, rendering their attribution as caniniform obsolete.

The aim of this study, based on an analysis of the type series and additional freshly preserved specimens, is to redescribe *O*. *xinguense* with attention to anatomical novelties (particularly osteological ones) and allometry between juveniles and adults as well as males and females. In addition, we provide important updates on the geographical distribution of this endangered and poorly known species of rheophilic fish, and cast perspectives on its conservation.

## Materials and Methods

Redescription and analysis based on the holotype, four paratypes, and more than 200 additional specimens ranging from 36.4 to 228.6 mm standard length (SL). Measurements and counts follow Jégu [1] and Andrade *et al*. [10]. Body measurements are given as percents of SL, and head subunits as percents of head length (HL). Counts are followed by the frequency in parentheses with the value observed in holotype indicated by an asterisk. Osteological descriptions based on two juvenile specimens cleared and stained (c&s) according to Taylor and Van Dyke [11], three adult specimens skeletonized (skel.), and radiograph of one paratype. Counts of vertebrae included the Weberian apparatus as four and the fused PU1+U1 caudal vertebrae as one. Osteological nomenclature follows Weitzman [12] with modifications proposed by Mattox *et al*. [13] and the addition of abdominal serrature composed of midventral spines oriented caudally (characteristic of Serrasalmidae taxa). Morphological comparisons employ previous observations in studies by Jégu [5], Machado-Allison [14], Dahdul [15], and Ota [16].

Variation in body shape was evaluated using principal components analysis (PCA) on 33 linear measurements according to Strauss [17]. Principal components were computed from the covariance matrix of log-transformed data according to Jolicoeur [18]. The first principal component (PC1) was interpreted as a general size factor (i.e., overall body size correlated significantly and positively with PC1) [19]. Analysis of covariance (ANCOVA) was used to compare the allometric trajectory of body shape between groups; analysis of variance (ANOVA) compared the means of body shape with respect to size class and sex.

Institutional abbreviations are ANSP (Academy of Natural Sciences of Drexel University, Philadelphia), GEA (Laboratório de Ictiologia do Grupo de Ecologia Aquática, Universidade Federal do Pará, Belém), INPA (Instituto Nacional de Pesquisas da Amazônia, Manaus), LIA (Laboratório de Ictiologia de Altamira, Universidade Federal do Pará, Altamira), MNHN (Muséum national d'Histoire naturelle, Paris), MPEG (Museu Paraense Emílio Goeldi, Belém), ZSM (Zoologische Staatssammlung München, München), and ZUEC (Museu de Zoologia da Universidade Estadual de Campinas ‘Adão José Cardoso’, Campinas).

## Results

*Ossubtus* Jégu 1992

*Ossubtus*.–Jégu [1]: 235–252 (original description).–Jégu [3]: 187 (catalog of freshwater fishes of South and Central America).–Jégu *et al*. [8]: 101 (taxonomic history of *Tometes*).–Dahdul [15]: 50 (phylogenetic comparisons based on previous studies).–Ota [16]: 28 (comments on *Myleus* clade members).–Jégu and Santos [20]: 51 (teeth compared to *Myleus*).–Jégu *et al*., [21]: 849 (*Myleus* group).–Zanata and Vari [22]: 29 (comparisons in phylogenetic analysis of family Alestidae).–Freeman *et al*. [23]: 3 (Serrasalmidae diversity).–Ortí *et al*. [4]: 346 (*Myleus* clade members).–Cione *et al*. [24]: 356 (tooth morphology compared to *Megapiranha*).–Mirande [25]: 6 (genera of the superfamily Characoidea).–Mirande [26]: 471 (node 187, Serrasalmidae).–Albert *et al*. [27]: 46 (examples of ostariophysan fishes).–Carvalho Jr. *et al*. [28]: 135 (ornamental fish of the Yudjá tribe).–Thompson *et al*. [29]: 2 (molecular phylogeny of Serrasalmidae with comparisons of methods).–Lujan and Conway [30]: 113 (specializations in rheophilic fishes).

### Diagnosis

*Ossubtus* is diagnosed from all members of Serrasalmidae by possessing a subinferior to inferior mouth (versus mouth terminal, or gently to distinctly upturned). It is distinguished from all Serrasalmidae except *Acnodon* by absence (versus presence) of spines on prepelvic serrae. It can be further distinguished from other herbivorous Serrasalmidae except *Myleus*, *Tometes* and *Mylesinus* by having incisiform teeth (Figs 1 and 2A–2C; versus molariform teeth, Fig 2D), and labial row of premaxillary teeth in contact with lingual row (versus labial and lingual rows of premaxillary teeth separated by gap). It can be distinguished from *Myleus*, *Tometes* and *Mylesinus* by having four teeth (versus five or more) on dentary, and the first two labial premaxillary teeth with crown reduced, narrow (*versus* first two labial premaxillary teeth with crown well-developed, width approximating tooth base). It also differs from *Myleus* and *Tometes* by having incisiform teeth very fragile, much flattened anteroposteriorly, and weakly attached to jaws (versus incisiform teeth robust and strongly attached to jaws).

*Ossubtus xinguense* Jégu 1992

Figs 1 and 3–13, Tables 1 and 2.

*Ossubtus xinguense*.–Jégu [1]: 235–252 (original description).–Jégu and Zuanon [2]: 414, figure 1 (conservation status).–Jégu [3]: 187 (species list).–Ortí *et al*. [4]: 346 (molecular phylogeny of Serrasalmidae).–Jégu [5]: 350 (synapomorphies of *Myleus* clade in phylogeny of subfamily Serrasalminae).–Andrade *et al*. [6]: 304 (identification key, comparative material).–Jégu *et al*. [8]: 199 (comparative material).–Andrade *et al*. [10]: 25 (comparative material).–Dahdul [15]: 10 (character descriptions in phylogeny of subfamily Serrasalminae).–Ota [16]: 312 (character descriptions in phylogeny of *Metynnis*).–Freeman *et al*. [23]: 38 (material examined in molecular systematic study).–Cione *et al*. [24]: 358 (comparative material).–Mirande [26]: 471 (morphological phylogeny of Characidae).–Lujan and Conway [30]: 113–114, figure 2i (specializations in rheophilic fishes, figure of mouth in ventral view).–Thatcher [31]: 293 (description of *Anphira xinguensis*, parasite of *Ossubtus*).–Zuanon [32]: 18 (species list, natural history).–Lundberg *et al*. [33]: 35, figure 9a (dietary specialists among Neotropical fishes).–Jégu *et al*. [34]: 273 (identification key, comparative material).–Jégu *et al*., [35]: 157 (comparative material).–Buckup *et al*. [36]: 41 (Catalog of Brazilian freshwater fishes).–Zuanon and Ferreira [37]: 28 (feeding ecology).–Zuanon and Jégu [38]: 87–88 (conservation status).–Camargo *et al*. [39]: 281 (figure in appendix 1).–Carvalho *et al*. [40]: 18 (examples of rapids-dwelling fishes).–Buckup and Santos [41]: 3–5 (fishes from Xingu-Tapajós ecoregion).–Camargo *et al*. [42]: 189 (commercial fishes from Xingu-Tapajós ecoregion).–Andrade *et al*. [43]: 102-104(biological parameters).–Giarrizzo *et al*. [44]: 6–9 (biological parameters).

*Ossubtus xinguensis* [mispelling].–Camargo *et al*. [45]: 135 (geographic distributions of Rio Xingu fishes).–Thatcher [46]: 422 (parasitology, isopod checklist).–Staeck [47]: 50 (aquarium hobby).–Menezes *et al*. [48]: 28, figure 6 (project newsletter, SACI—South American Characiformes Inventory).

#### Material examined

Holotype. ‒INPA 6535, female, 170.2 mm SL, Brazil, Pará, Altamira, Rio Xingu, 3°12'12"S 52°12'23"W, 31 Oct 1990, Mr. Izaltino Barbosa.

Paratypes. ‒MNHN 1992–0003, 1, 39.7 mm SL; MNHN 1992–0004, 2, 156.2–173.0 mm SL; ZSM 27765, 4, 51.0–176.0 mm SL (plus radiograph of largest specimen), same data as holotype.

#### Non-type material examined

All from Brazil, Pará. ‒GEA 1729, 130, 149.4–219.3 mm SL; GEA 1999, 2 skel., 181.7–183.7 mm SL; and INPA 48826, 1 skel., 188.1 mm SL, Altamira, Rio Xingu, rapids near ilha da Taboca, approximately at 3°22'04"S 51°59'59"W, Jul 2012, M. C. Andrade and T. Giarrizzo. GEA 1973, 1, 202.7 mm SL, Altamira, Rio Xingu, Cachoeira do Espelho, 3°40'08.2"S 52°26'17.8"W, 4 Jul 2012, L. M. Sousa. GEA 2276, 1, 228.6 mm SL, Altamira, Rio Xingu, Pedral do Reboque Velho, 3°22'05''S 51°59'59.3''W, 27 Jul 2011, M. C. Andrade. GEA 2277, 31, 44.2–129.0 mm SL, Altamira, Rio Xingu, Cachoeira do Jericoá, 3°21'51.7"S 51°43'59.0"W, 19 Jul 2014, L. M. Sousa. GEA 2278, 1, 76.8 mm SL, Cachoeira Grande do Rio Iriri, 3°50'38.3"S 52°44'02"W, 14 Sep 2012. L. M. Sousa. GEA 2279, 6, 55.1–72.2 mm SL, same locality as GEA 2277, 8 Jul 2012, L. M. Sousa. GEA 2280, 7, 61.2–80.9 mm SL, same locality as GEA 2277, 21 Sep 2012, L. M. Sousa. INPA 6536, 1, 53 mm SL (c&s), Senador José Porfírio, Rio Xingu, Cachoeira do Kaituká, approximately 3°33'47.0"S 51°53'20.0"W, 9 Oct 1990, L. Rapp Py-Daniel and J. Zuanon; INPA 38073, 7, 60.7–78.2 mm SL (1 c&s 60.7 mm SL), Altamira, Rio Xingu, Praia do Cajú near Cachoeira do Jericoé, 3°22'56"S 51°44'11"W, 13 Oct 2012, M. Sabaj Pérez, L.M. Sousa and M. Arces. ‒LIA 2987, 7, 36.4–68.9 mm SL; and LIA 2992, 10, 45.0–60.8 mm SL, Vitória do Xingu, Rio Xingu, Cachoeira de Jericoá, 3°19'29.0"S 51°44'53.5"W, 19 Jul 2013, A. P. Gonçalves. LIA 2988, 2, 38.9–41.4 mm SL, Altamira, Rio Xingu, 3°21'54.2"S 52°01'38.0"W, 23 Oct 2013, A. P. Gonçalves. LIA 2989, 1, 60.1 mm SL; LIA 2991, 4, 64.8–70.6 mm SL; LIA 2994, 19, 49.1–75.5 mm SL, same locality as LIA 2987, 21 Oct 2013, A. P. Gonçalves. LIA 2990, 1, 91.1 mm SL; LIA 2993, 2, 67.9–74.5 mm SL, Anapú, Rio Xingu, downstream of UHE Belo Monte powerhouse, 3°06'45.5"S 51°43'10.6"W, 26 Oct 2013, A. P. Gonçalves. ‒MNHN 1998–1168, 4, 36.4–55.9 mm SL, same locality as INPA 6536, M. Jégu, 10 Oct 1990; ‒MPEG 30686, 25, 45.6–132.5 mm SL, Volta Grande do Rio Xingu, 3°19'30.4"S 51°44'54.9"W, 8 Jul 2012. L. M. Sousa. MPEG 30690, 2, 56.8–114.6 mm SL, same locality as GEA 2278, 3 Jul 2012. L. M. Sousa. ‒ZUEC 11532, 1, 190.2 mm SL, same locality as GEA 1973, 3 Jul 2012. L. M. Sousa. ZUEC 11533, 1, 173.2 mm SL, Altamira, Rio Iriri, Cachoeira Grande, 3°50'37.4"S 52°44'08.5"W, 14 Sep 2012. L. M. Sousa.

### Diagnosis

Same as generic diagnosis.

### Description

**Fig 3:**
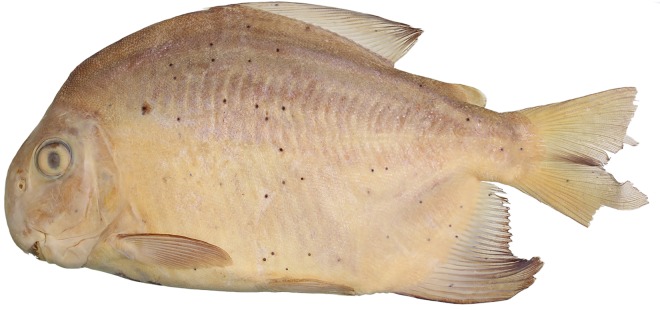
*Ossubtus xinguense*, holotype, INPA 6535, female, 170.2 mm SL, Brazil, Rio Xingu at rapids downstream Altamira.

**Fig 4:**
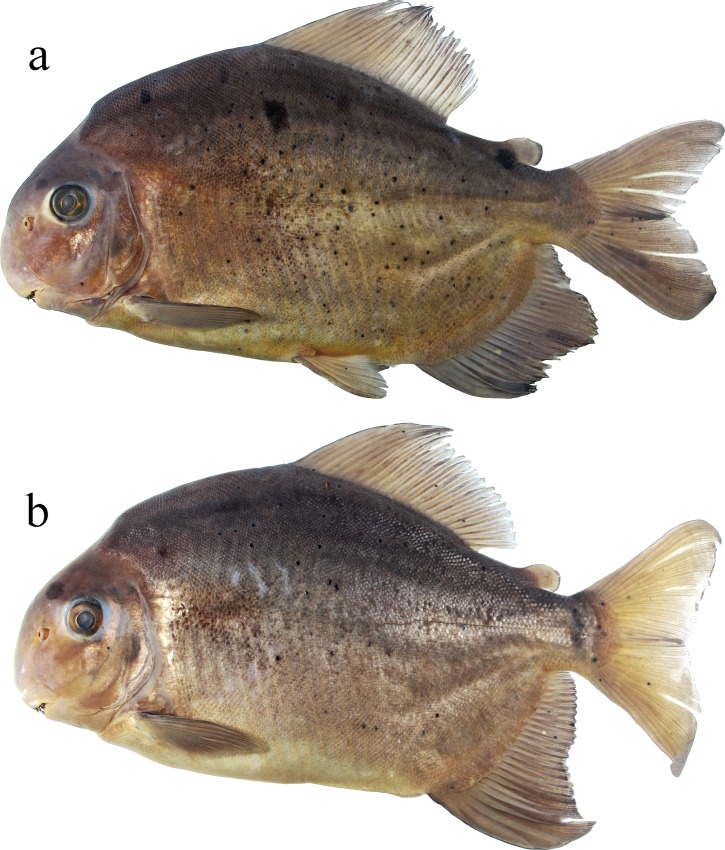
Adults of *Ossubtus xinguense*: GEA 1973, (a) male, 202.7 mm SL; (b) female, 168.5 mm SL. Brazil, Rio Xingu, Volta Grande.

**Fig 5:**
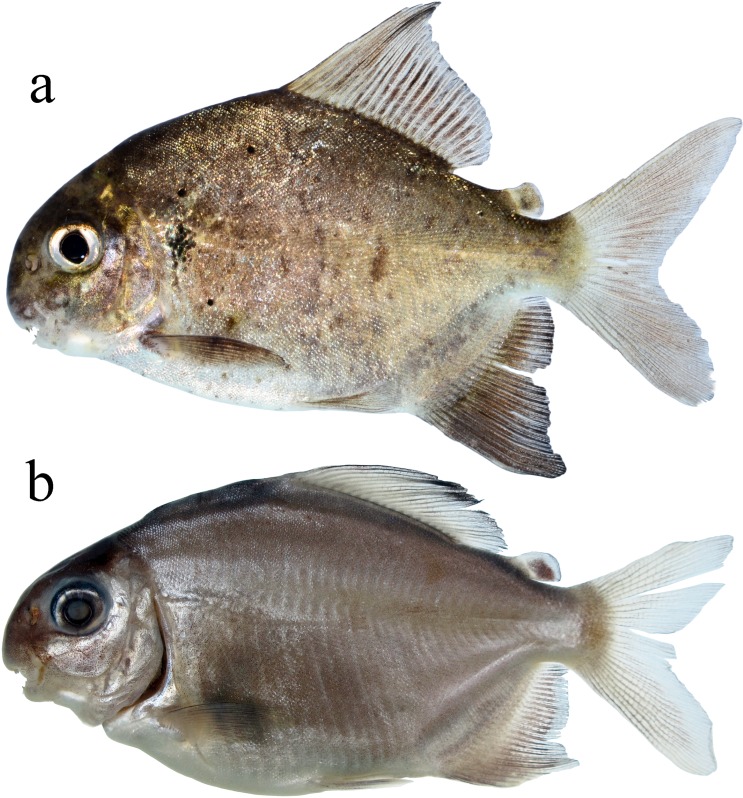
Juveniles of *Ossubtus xinguense*: (a) MPEG 30686, 66.4 mm SL (photographed alive), (b) 45.6 mm SL (preserved).

Morphometric data presented in Table 1. Medium sized serrasalmid, largest examined specimen 228.6 mm SL. Body laterally compressed, profile sub-ovoid (Figs 3–5). Predorsal profile steep. Dorsal profile of head markedly convex from upper lip to vertical through anterior nares, gently straight or nearly concave from that point to distal margin of supraoccipital spine, and slightly convex from that point to dorsal-fin origin. Greatest body depth at dorsal-fin origin, means 56.7% of SL in adults and 53.8% SL in juveniles. Dorsal-fin base straight to gently convex. Body profile straight from dorsal-fin terminus to adipose-fin origin. Ventral profile of head straight to gently convex. Ventral body profile distinctly convex. Anal-fin base convex. Caudal peduncle short, upper and lower profiles concave.

**Table 1:** Morphometric characteristics of *Ossubtus xinguense* (n = 216). Range includes the type specimens. Hol = Holotype, SD = Standard Deviation. Measurements 1–23 are expressed as percentages of standard length, and 24–33 as percentages of head length.

	Hol	*Adults* (n = 148)		*Juveniles* (n = 68)	
		Range	Mean ± SD	Range	Mean ± SD
Standard length (mm)	170.2	114.5–228.6	176.3	36.4–92.1	60.7
1. Body depth	53.8	48.0–63.9	56.7 ± 2.6	48.9–60.0	53.8 ± 2.1
2. Head length	26.1	22.7–28.5	26.4 ± 0.9	25.4–29.4	26.8 ± 0.7
3. Supraoccipital process	36.3	30.4–38.6	35.0 ± 1.2	30.4–37.7	33.6 ± 1.4
4. Predorsal length	59.1	53.6–63.3	59.8 ± 1.4	52.3–58.8	55.7 ± 1.2
5. Dorsal-fin base length	33.5	28.7–38.0	35.0 ± 1.2	27.4–36.1	32.9 ± 1.4
6. Interdorsal length	11.9	8.53–12.9	10.8 ± 0.8	7.1–10.8	9.1 ± 0.8
7. Adipose-fin base length	4.6	2.9–5.1	4.0 ± 0.3	3.1–5.1	4.4 ± 0.3
8. Caudal-peduncle depth	12.9	11.3–13.8	12.7 ± 0.4	10.7–13.3	12.0 ± 0.5
9. Anal-fin base length	26.8	25.3–31.2	27.9 ± 1.0	23.0–28.7	26.1 ± 1.2
10. Preanal length	71.9	62.6–75.3	70.9 ± 1.7	70.6–77.7	74.1 ± 1.6
11. Prepelvic length	48.5	43.7–52.2	48.6 ± 1.3	50.4–58.9	53.9 ± 1.7
12. Prepectoral length	20.6	18.1–22.7	20.5 ± 0.9	21.6–29.9	24.7 ± 2.1
13. Anal-pelvic distance	23.9	19.0–25.3	22.6 ± 1.2	19.5–24.1	21.6 ± 1.0
14. Pelvic-pectoral distance	29.9	25.7–33.0	29.5 ± 1.1	27.3–33.6	30.4 ± 1.2
15. Width of peduncle	3.9	2.7–4.4	3.4 ± 0.3	2.5–4.3	3.2 ± 0.3
16. Pectoral-fin length	23.8	19.5–27.2	23.5 ± 1.0	21.3–25.6	23.9 ± 0.9
17. Pelvic-fin length	18.1	15.6–23.5	18.0 ± 1.1	15.6–19.7	17.4 ± 0.8
18. 1st anal-fin lobe length	28.7	17.5–34.6	27.2 ± 3.3	19.0–29.2	25.3 ± 2.1
19. 2nd anal-fin lobe length	*	12.7–28.3	19.6 ± 3.0	*	*
20. Dorsal-fin length	26.2	23.1–37.8	31.1 ± 2.8	22.6–34.0	28.8 ± 2.5
21. Distance from dorsal-fin origin to anal-fin origin	61.7	54.3–68.5	63.5 ± 2.4	52.7–63.4	58.2 ± 2.3
22. Distance from dorsal-fin end to anal-fin origin	41.7	37.3–48.0	42.9 ± 1.8	36.0–43.9	39.8 ± 1.6
23. Distance from dorsal-fin end to anal-fin end	26.2	21.6–27.5	25.3 ± 1.0	21.7–26.2	23.9 ± 0.8
24. Snout length	52.2	43.7–57.3	49.8 ± 1.9	28.7–42.3	37.3 ± 2.9
25. Interorbital width	45.2	39.7–47.6	43.5 ± 1.5	26.5–45.0	37.4 ± 3.2
26. Head width	63.0	52.2–65.9	58.5 ± 2.5	50.1–66.0	59.2 ± 3.4
27. Postorbital distance	24.0	20.3–28.2	25.4 ± 1.3	23.6–32.5	26.8 ± 1.9
28. Fused 4th infraorbital width	7.4	7.3–12.9	10.8 ± 1.0	8.6–15.8	12.0 ± 1.5
29. Eye vertical diameter	34.3	25.5–35.7	30.8 ± 1.7	30.2–39.9	35.5 ± 2.2
30. Mouth length	17.1	11.3–18.9	15.9 ± 1.3	10.0–18.7	14.2 ± 1.9
31. 3rd infraorbital width	18.4	13.4–21.6	17.3 ± 1.6	7.8–13.6	10.6 ± 1.4
32. Cheek gap width	14.2	11.3–18.2	14.6 ± 1.2	13.7–19.6	16.8 ± 1.3
33. Mouth width	35.8	30.9–39.6	34.8 ± 1.5	25.6–33.7	30.4 ± 1.4

Mouth subterminal to subinferior in juvenile specimens up to 50 mm SL (Fig 5B), and markedly inferior in larger specimens. Snout strongly rounded. First branchial arch with gill rakers elongated and recurved. Gill rakers in upper branch 10 (1), 11 (6), or 13 (2), and in lower branch 13 (1), 14 (5), or 15 (4); one gill raker at cartilage between upper and lower branches.

Body fully covered with cycloid scales. Base of dorsal and anal fins covered by scaly sheath; dorsal fin with 2* (21) scale rows in sheath and anal fin with 6 (1), 7 (18), or 8* (2) scale rows in sheath. Lateral line complete with imbricate scales on supracleithrum; scales from supracleithrum to hypural joint 73 (1), 74 (1), 75 (4), 77 (1), 78 (1), 79* (8), 80 (5), 81 (5), 82 (2), 83 (2), 84 (2), 85 (1), or 86 (1) scales; total perforated scales 78 (1), 80 (4), 82 (1), 83 (1), 84* (5), 85 (4), 86 (6), 87 (5), 88 (3), 89 (1), 90 (2), or 92 (1). Scale rows between dorsal fin origin and lateral line 40 (1), 41 (1), 42* (2), 43 (1), 44 (1), 45 (5), 46 (4), 47 (7), 48 (2), 49 (3), 50 (3), 51 (2), or 53 (2). Scale rows between lateral line and pelvic fin insertion 37 (1), 38 (1), 39 (3), 40 (7), 41* (2), 42 (5), 43 (3), 44 (4), 45 (1), 46 (3), 47 (2), 49 (1), or 50 (1). Scales rows around caudal peduncle 36 (4), 37 (3), 38* (9), 39 (7), 40 (5), 41 (3), 42 (1), or 43 (2). Abdominal prepelvic region lacking spines in all examined specimens. Postpelvic serrae with 6 (5), 7* (22), or 8 (7) simple spines, and 4 (2), 5 (12), 6 (14), 7* (5), or 8 (1) pairs of spines around anus. Total postpelvic spines 11 (4), 12 (11), 13 (8), 14* (8), or 15 (3).

Distal margin of dorsal fin clearly falcate in juveniles up to 65 mm SL, somewhat straight in specimens greater than 80 mm SL. Dorsal-fin origin preceded by strong anteriorly directed procumbent spine covered with skin. Dorsal-fin rays ii (4), iii* (22), or iv (9), and 19 (2), 20* (6), 21 (15), or 22 (11). Adipose-fin base short, distal lobe well-developed, directed posteriorly, margin squarish to completely rounded. Pectoral-fin rays i (34), 15 (12), 16* (13), or 17 (9). Pelvic-fin rays i, 7* (34). Anal-fin rays iii* (31), or iv (3), and 23* (8), 24 (18), or 25 (8). Distal margin of anal fin sexually dimorphic, falcate in females (Figs 3 and 5B) and juveniles (Fig 5); males exhibit second lobe formed by middle rays (Fig 4A, see also under Sexual dimorphism). Caudal fin moderately forked, lobes rounded and similarly sized.

Five* (6) supraneurals, with 1st and 5th supraneurals positioned anterior to neural spine of 4th* and 8th* centra, respectively. First dorsal-fin pterygiophore inserted posterior of neural spine of 10th* (6) centrum. First anal-fin pterygiophore inserted behind haemal spine of 22nd* (6) centrum. Thirty-eight* (6) total vertebrae, with 19* (6) precaudal and 19* (6) caudal vertebra.

#### Osteology

**Fig 6:**
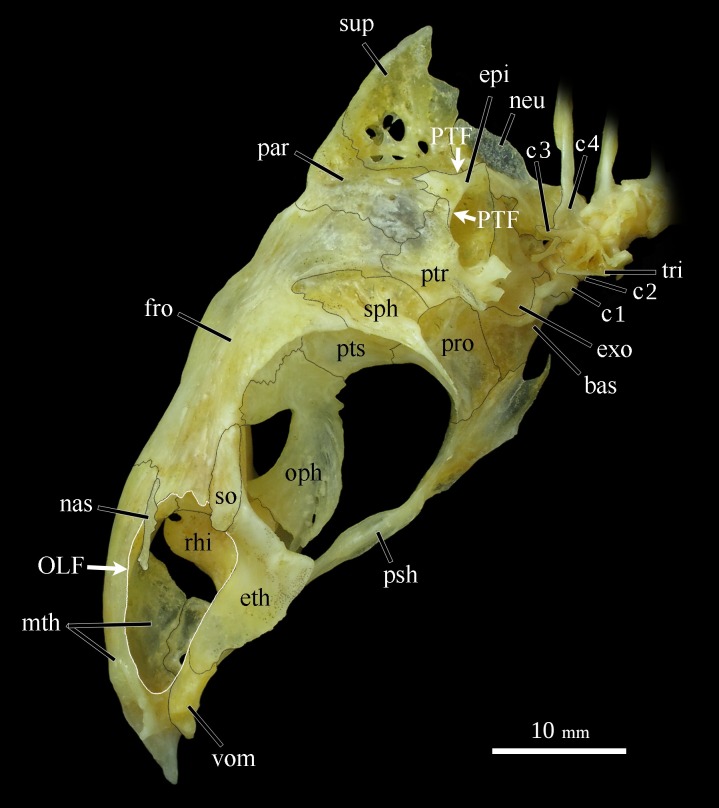
Left lateral view of neurocranium in *Ossubtus xinguense*, GEA 1999, female, 181.7 mm SL, oriented in natural position; c1-4 Compound centra 1–4, bas Basioccipital, epi Epiotic, eth Lateral Ethmoid, exo Exoccipital, fro Frontal, mth Mesethmoid, nas Nasal, neu Neural Complex, oph Obitosphenoid, par Parietal, pro Prootic, psh Parasphenoid, ptr Pterotic, pts Pterosphenoid, rhi Rhinosphenoid, sph Sphenotic, so Supraorbital, sup Supraoccipital, tri Tripus, vom Vomer. OLF Olfactory fossae (white outline), PTF Posttemporal fossae.

**Fig 7:**
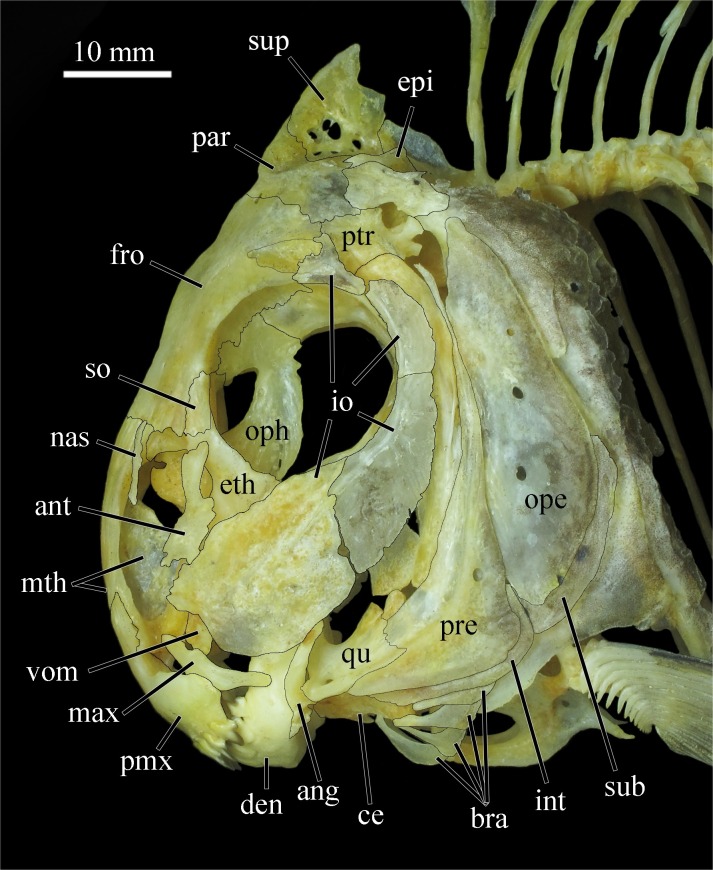
Head in *Ossubtus xinguense*, GEA 1999, male, 183.7 mm SL; ang Anguloarticular, ant Antorbital, bra Branchiostegal Rays, ce Ceratohyal, den Dentary, epi Epiotic, eth Lateral Ethmoid, fro Frontal, int Interopercle, io Infraorbital Series, max Maxilla, mth Mesethmoid, nas Nasal, ope Opercle, oph Obitosphenoid, par Parietal, pmx Premaxilla, pre Preopercle, ptr Pterotic, qu Quadrate, sub Subopercle, so Supraorbital, sup Supraoccipital, vom Vomer.

Neurocranium. overall neurocranium shallow and elongated with slender bones; long axis set at about a 45° angle from longitudinal axis of body (Figs 6 and 7).

Olfactory region: composed by mesethmoid, lateral ethmoid, vomer, and nasal (Fig 6). Mesethmoid triangular in frontal view, profile gently curved with ventral portion (lateral wings) finishing vertically; posteriorly directed keel large with anteroventral portion pointed, surpassing lateral wings and vomer. Mesethmoid with posterolaterally and posteroventrally portions not contacting lateral ethmoid. Lateral ethmoid elongated, in contact dorsally with anteroventral terminus of frontal; lateral wing thin, pointed distally, and directed ventrally. Anteromedial process of mesethmoid reaching posterior portion of vomer. Vomer participating in posterior rim of wide olfactory fossa. Nasal long and narrow with well-defined canal.

Orbital region: composed by parasphenoid, frontal, pterosphenoid, and orbitosphenoid (Fig 6). Parasphenoid long with ventral aperture forming two thin projections somewhat parallel across ventral margins of prootic and basioccipital. Parasphenoid contacts prevomer anteriorly, prootic dorsally and basioccipital posteriorly. Frontal large, subrectangular, length approximately 50% of neurocranium length; lateral margin of posterior half with deep notch receiving sphenotic; superficial surface with foraminas and grooves. Cranial fontanel elongated, narrower at extremities; portion anterior to epiphyseal bar almost two times longer than posterior portion. Pterosphenoid laminar, laterally articulated with sphenotic. Orbitosphenoid deep, composed of two laterally compressed bony lamellae projecting posteroventrally, but not contacting parasphenoid; anterior process enlarged with anterodorsal surface in contact with internal surface of frontal.

Otic region: composed by prootic, sphenotic, parietal, intercalar, pterotic, and epiotic (Fig 6). Prootic quadrangular, with circular aperture for myodome passage. Sphenotic narrow with concave margin contributing to orbit, finishing posterolaterally with long pointed spine for origin of *dilator operculi* muscle. Parietal short and wide, increasing in width laterally; dorsal process well developed with surface sculptured by grooves. Intercalar with well-developed posterolateral portion, located in posterior region of neurocranium. Pterotic with short process ending in two truncated lobes posteriorly directed. Epiotic with lateral arm extending towards posterior margins of parietal and pterotic, dividing posttemporal fossa into dorsal and ventral portions (Fig 6).

Occipital region: composed by basioccipital, exoccipital, and supraoccipital (Fig 6). Basioccipital forming entire ventral surface of saccular capsule. Exoccipital surrounding lateral occipital foramen, lagenar capsule well developed. Supraoccipital spine relatively short, dorsal profile gently convex, distal portion triangular with rounded point.

Infraorbital series: composed by antorbital, infraorbitals 1–4, and supraorbital (Fig 7). Antorbital with ventral half larger, directed anteroventrally and reaching fourth infraorbital; dorsal half narrower, nearly vertical. First infraorbital well developed, overall size slightly smaller than circular orbit; anterodorsal margin (anterior to contact with antorbital) with shallow, irregular crenations; laterosensory canal obliquely oriented near center of bone. Second infraorbital smaller, wing shaped (ventral portion expanded), obliquely oriented with laterosensory canal slightly removed from dorsal margin. Third infraorbital vertically elongated with laterosensory canal close to anterior margin. Fourth infraorbital smallest, with anterodorsal portion expanded, forming obliquely oriented “Y” with irregular margin sutured to frontal; laterosensory canal restricted to posteroventral portion. Supraorbital subrectangular, contributing to orbit rim but not contacting antorbital, leaving small gap in circumorbital series.

**Fig 8:**
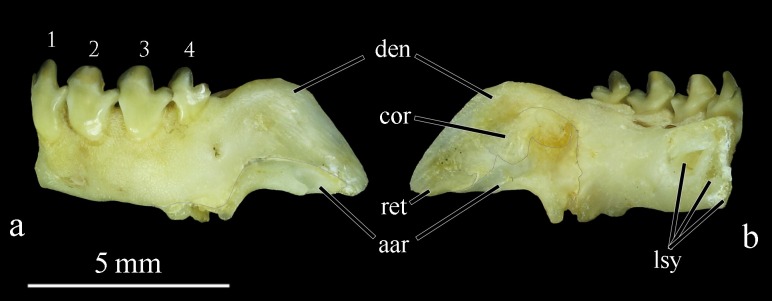
Dentary of *Ossubtus xinguense*, GEA 1999, female, 181.7 mm SL, (a) lateral view, (b) lingual view; aar Anguloarticular, cor Coronomeckelian, den Dentary, lsy Lamellae at Symphysis, ret Retroarticular, 1–4 Dentary teeth.

Jaws: premaxilla high with wide surface; interdigitations lacking at symphyseal sututre, Ascending premaxilla process elongated, slender, and oblique. Lateral premaxilla process short, subrectangular. Premaxilla with two rows of fragile, weakly inserted, incisiform teeth. Teeth visible outside mouth, labial row in internal contact with lingual row. Premaxilla labial row with five teeth and lingual with two teeth. First and second teeth of labial row with poorly-developed edges; three remaining teeth well developed, high, trilobed and spatulate (Fig 1A). First premaxilla lingual teeth bilobed and second trilobed (Fig 1B). Teeth in juvenile specimens up to 70 mm SL with narrow cusps, higher, with main cusp resembling spearhead; teeth of adults with rounded cusp edges, lower, with main cusp resembling flattened spoon. Maxilla edentulous, narrow, with middle expansion connected to posterior arm of premaxilla. Paired symphyseal teeth absent from dentary. Posterodorsal margin of dentary convex with apex not reaching horizontal through tip of fourth tooth; posterior margin sloped at 45° angle with long axis of lower jaw. Dentary with four teeth, first trilobed, second to fourth bilobed (Fig 8A). Dentary teeth with posterior cusp of each tooth inserted externally into groove of anterior cusp of next tooth. Symphyseal dentary teeth absent. Retroarticular elongated, lenticular, reaching ventral margin of lower jaw but slightly removed its posteroventral tip completed by dentary. Anguloarticular elongated, articulated with quadrate by thin cartilage. Cononomeckelian comma shaped. Dentary symphysis with three bony lamellae oriented obliquely to long axis of bone (Fig 8B).

**Fig 9:**
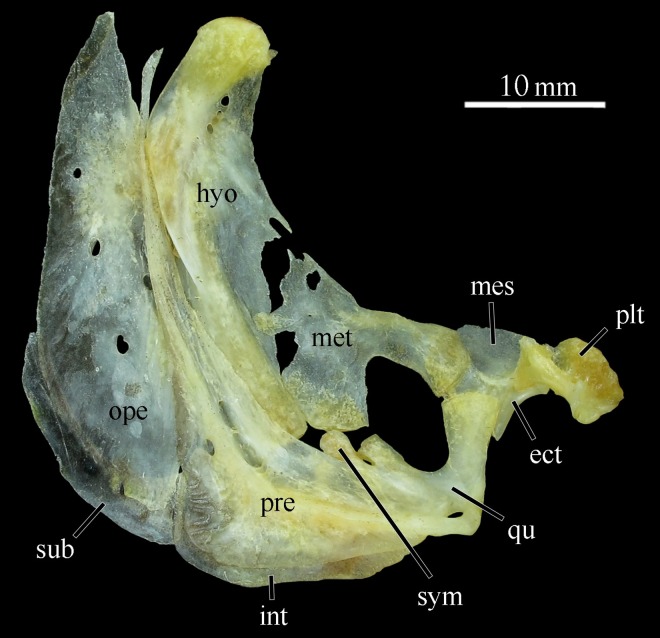
Hyopalatine arch of *Ossubtus xinguense*, GEA 1999, female, 181.7 mm SL in lateral view (anterior is right); ect Ectopterygoid, hyo Hyomandibular, int Interopercle, mes Mesopterygoid, met Metapterygoid, ope Opercle, plt Autopalatine, pre Preopercle, qu Quadrate, sub Subopercle, sym Symplectic.

Hyopalatine arch: composed by quadrate, hyomandibular, endopterygoid, symplectic, ectopterygoid, metapterygoid and autopalatine (Fig 9). Ascendent process of quadrate perpendicular to longitudinal body axis, oblique to long axis of dentary. Hyomandibular narrow and elongated; anterior process curved, posterior process oblique. Endopterygoid subquadrangular, with anteroventral margin along with dorsal margins of ectopterygoid and quadrate. Sympletic small, extending posterodorsally into metapterygoid-quadrate fenestra. Ectopterygoid narrow and oblique, anterior margin with concavity, overall shape like hour glass, ecto-endopterygoid articulation anterior of posterior margin of autopalatine. Metapterygoid laminar, “T” shaped, with dorsal surface oblique to articulation of endopterygoid. Metapterygoid anteroventrally articulated with quadrate and posteroventrally with hyomandibular. Autopalatine hemicylindrical, relatively short, horizontally and anteriorly directed.

Opercular series: composed by opercle, interopercle, subopercle, and preopercle (Fig 9). Opercle laminar, narrow and vertically elongated, lenticular with ventral portion slightly wider than dorsal, and anterior margin sculpted with grooves. Interopercle narrowly subtriangular, posterior portion with ascending process occupying preopercle-subopercle fenestra. Subopercle narrowly elongated, curved along posteroventral margin of opercle. Preopercle laminar and curved along posteroventral margins of quadrate and hyomandibular; anterior lamellar flange extending almost to hyomandibular-opercle condyle; median laterosensory canal alongside flange gently curved.

Hyoid arch: composed by branchiostegal rays, anterior ceratohyal, urohyal, ventral hypohyal, posterior ceratohyal, dorsal hypoyal, basihyal, and interyal. Four short branchiostegal rays (Fig 7), fourth longest; first and second rays attached to expanded ventral area at posterior third of anterior ceratoyal; third and fourth rays attached to ventral area of anterior ceratohyal. Anterior certaohyal subrectangular with ventral portion larger than dorsal. Urohyal bearing thin dorsal flange, giving bone triangular shaped in lateral view. Dorsal hypohyal well developed with anterior portion expanded, surpassing vertical through anterior and posterior ceratohyal articulation. Hypohyal not symmetrical with ventral hypohyal smaller than dorsal hypohyal. Urohyal attached at anterior margin of ventral hypohyal. Posterior ceratohyal triangular with base attached to ventral margin of anterior ceratohyal. Interhyal small, narrow and articulated to posteroventral portion of posterior certatohyal. Basihyal narrow with dorsal portion larger than ventral.

Branchial skeleton: composed by ceratobranchials 1–5, epibranchials 1–5, pharyngobranchials 1–5, basibranchials 1–3, and hypobranchials 1–3. First to 5th ceratobranchials with 10–12 thin gill rakers. First to 4th epibranchial ossified with 10–12 think, short gill rakers. Fifth epibranchial cartilaginous, even in adults. First to 4th pharyngobranchial ossified; 5th pharyngobranchial cartilaginous. First to 3rd basibranchial thin and ossified. First to 3rd hypobranchial lacking gill rakers.

Weberian apparatus and associated centra: composed by compound centra 1–4, neural arches 3 and 4, neural spine of vertebra 4, intercalarium, scaphium, inner and outer arms of os suspensorium, tripus, claustrum, and neural complex. Centra 1 and 2 of approximately equal size (Fig 6). Lateral process of centrum 2 well-developed, longitudinally elongated, articulated ventrolateraly with centra 3. Neural arches 3 and 4 joined by thin suture; neural arch 3 small; neural arch 4 bearing elongated neural spine of 4th vertebra (Fig 6). Intercalarium thin, scaphium short, composed by concha scaphium and ascending process. Inner arm of os suspensorium as robust process posteriorly directed and parallel to dorsal surface of swimbladder. Outer arm of os suspensorium short and dorsoventrally flattened. Tripus aligned with intercalar and lateral process of 3rd centrum with ventral margin straight for centrum (Fig 6). Claustrum with short ascending arm, and bearing triangular flange along dorsal margin. Neural complex with posterior lamelar portion narrow (Figs 6 and 7).

Axial skeleton: composed by vertebral centra, neural arches, haemal spines, ribs, neural spines, parapophyses, and supraneurals. All vertebrae with neural arch and dorsal neural spine. Vertebral centra composed by 13 precaudal vertebrae (5th to 17th vertebrae) and 21 caudal vertebrae (18th to 38th vertebrae) including three pleural vertebrae and compound caudal centrum. Precaudal vertebrae lack haemal spines and bear parapophyses articulating with ribs. Caudal vertebrae have haemal arch with ventral haemal spine. Eighteenth and 19th vertebrae lack haemal spine, but with closed haemal arch bearing tiny ribs. Haemal spines of 20th and 21st vertebrae short.

**Fig 10:**
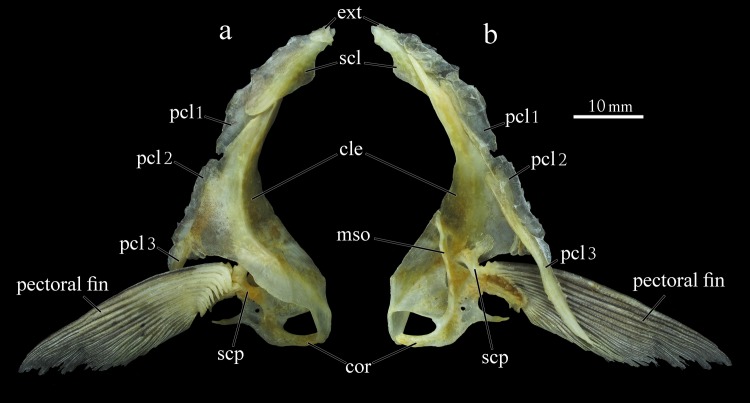
Pectoral girdle of *Ossubtus xinguense*, GEA 1999, female, 181.7 mm SL, (a) external view, (b) internal view; ext Extrascapular, cle Cleithrum, cor Coracoid, mso Mesocoracoid, scl Supracleithrum, scp Scapula, pcl1-3 Postcleithrum 1–3.

Pectoral girdle: composed by cleithrum, supracleithrum, posttemporal, extrascapular, postcleithrum 1–3, scapula, coracoid, mesocoracoid, and pectoral fin rays i,15–17 (Fig 10). Cleithrum well developed with pointed dorsal tip extending to middle portion of supracleithrum. Supracleithrum bearing relatively large posterior flange with dorsal tip articulating with posttemporal. Posttemporal bone with lateral line canal extending from dorsal tip to posteroventral margin. Posttemporal thin, oblique and slightly curved with pointed dorsal tip. Extrascapular lamelar, anteroventral to posttemporal. Postcleithrum 1 oval and vertically elongated with posterior portion lamelar, approximately half of anterior dorsal portion laterally overlapped by supracleithrum. Postcleithrum 2 vertically elongated with posterior portion lamelar, and anterior portion overlapped by cleithrum. Postcleithrum 3 elongated and pointed, extending beyond ventral margin of pectoral fin. Scapula with anterior process narrow, not covering space between scapula, cleithrum and coracoid, filled with thin connective tissue. Coracoid contacting cleithrum bilaterally with ventral margin curved. Mesocoracoid vertical with dorsal portion articulated to horizontal process of cleithrum, contacting coracoid ventrally.

**Fig 11:**
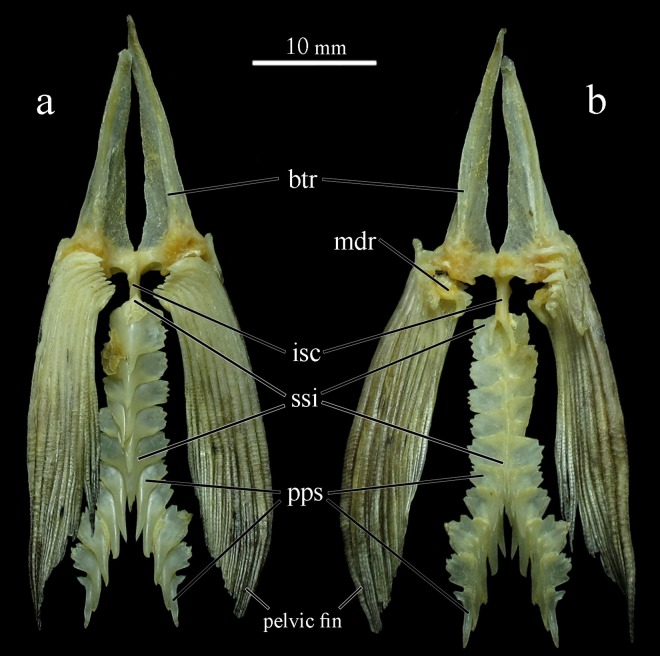
Pelvic girdle and abdominal serrature of *Ossubtus xinguense*, GEA 1999, female, 181.7 mm SL, (a) vetral view, (b) internal view; btr Basipterygium, isc Ischiatic, mdr Medial Radial, ssi Simple Postpelvic Spines, pps Pairs of Postpelvic Spines.

Pelvic girdle: composed by basipterygium, and pelvic fin rays i,7 (Fig 11). Basipterygium elongated with ischiatic process long and pointed, nearly reaching first third of outermost pelvic fin rays.

Abdominal serrature: composed by 6–9 simple postpelvic spines anteriorly, and 4–9 pairs postpelvic spines around anus, 11–16 spines total (Fig 11).

Dorsal fin: composed by proximal-middle radials, predorsal spine, and dorsal-fin rays ii-iv, 19–22. Proximal-middle dorsal-fin radials 21, each with lateral lamelar process. First proximal-middle dorsal fin radial modified, bearing continuous predorsal spine, anteriorly directed, lacking lateral processes, and dorsal margin evenly convex. Mature males with dorsal-fin rays prolonged as filaments (see details in Sexual dimorphism).

**Fig 12:**
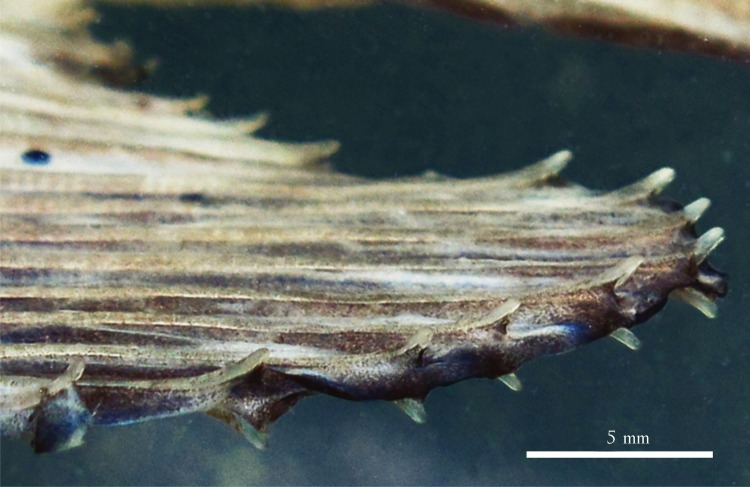
*Ossubtus xinguense*, GEA 1999, male, 183.7 mm SL. Ventrolateral view of the second anal-fin lobe in mature males, distal tip of each lepidotrichia with a pair of stiff hooks laterally curved.

Anal fin: composed by proximal-middle radials, and anal-fin rays iii-iv, 22–25. Proximal-middle anal fin radials 25, anterior three each with lateral lamelar process. Males with second pointed lobe formed by elongated middle rays, each one sometimes with pair of stiff laterally curved hooks near distal tip (Fig 12, see more details under Sexual dimorphism).

**Fig 13:**
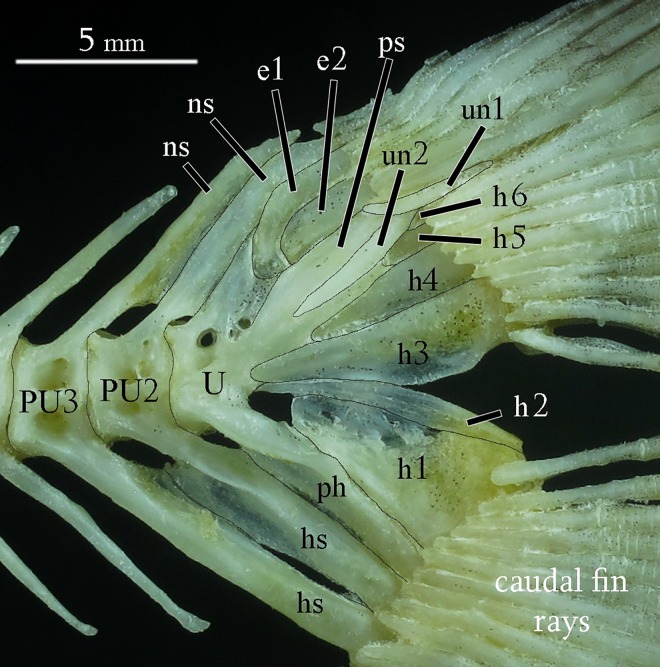
Caudal-fin skeleton of *Ossubtus xinguense*, GEA 1999, male, 183.7 mm SL; e1-2 Epural 1 and 2, h1-6 Hypurals 1 to 6, hs Haemal Spine, ns Neural Spine, ph Parhypural, PU2-3 Preural Centrum 2 and 3, U Compound Ural Centrum, un1-2 Uroneural 1 and 2.

Caudal fin and supporting skeleton: composed by epurals, haemal arches with spines of preural centra 2 and 3, hypurals 1–6, neural arches with spines of preural centra 2 and 3, parhypural, preural centra 2 and 3, principal caudal-fin rays 9+8, procurrent caudal-fin rays, ural and pleural centra 1, and uroneurals. Epural 1 anteriorly located, and epurals 1–2 spaced between neural arch and spine of preural centrum 3, pleurostyle and uroneural 2 (Fig 13). Haemal arches and spines of preural centra 2 and 3 well developed, narrow and fused. Hypural 1 well developed, hypural 2 narrow, hypural 3 horizontally directed, hypural 4 along with hypural 5 and 6 very small. Neural arches and spines of preural centra 2 narrow and elongated. Parhypural along with hypural 1. Uroneural 2 narrow along with margin of pleurostyle.

#### Color in alcohol

Background color silvery brown, head and upper flanks darker. Most specimens with lower flank light brown (Figs 3, 4 and 5B). Ventral surface of head, opercular region, supracleithrum, abdomen, and anal-fin base pale yellow. Irregular brownish blotches scattered over flank, mainly in mature males (Fig 4A). Larger adults with large diffuse blotch formed by few scattered melanophores on anterior-medial portion of flank (Fig 4). In juveniles, dorsal fin with concentration of melanophores on anterodistal margin, humeral blotch shaped as irregular inverted triangle, faint in smallest preserved specimens. Juveniles up to 60 mm SL with adipose fin pigmented (Fig 5B).

#### Color in life

Similar to that described for preserved specimens (Fig 5A), except ventral surface of body more pale. Adipose fin darker in specimens up to 60 mm SL, becoming hyaline with growth. Triangular humeral blotch iridescent turquoise, observable in specimens up to 130 mm SL.

#### Sexual dimorphism

Mature males of *O*. *xinguense* are recognized in specimens with 150 mm SL or more by exhibit additional anal-fin lobe formed by branched rays 12–14 (Fig 4A), whereas females have anal fin with falcate distal margin (Figs 3 and 4B). Some males with 180 mm SL or greater have pair of stiff, laterally divergent hooks near distal tip of each anal-fin ray in additional lobe (Fig 12). Stiff hooks on rays of additional lobe were observed in 36 of 52 mature male specimens greater than 180 mm SL. Dorsal-fin rays extended by modest filaments in 11 male specimens greater than 160 mm SL. All mature specimens with dark blotches on flanks (described under Color in alcohol), but blotches more intense in males.

#### Geographic distribution

**Fig 14:**
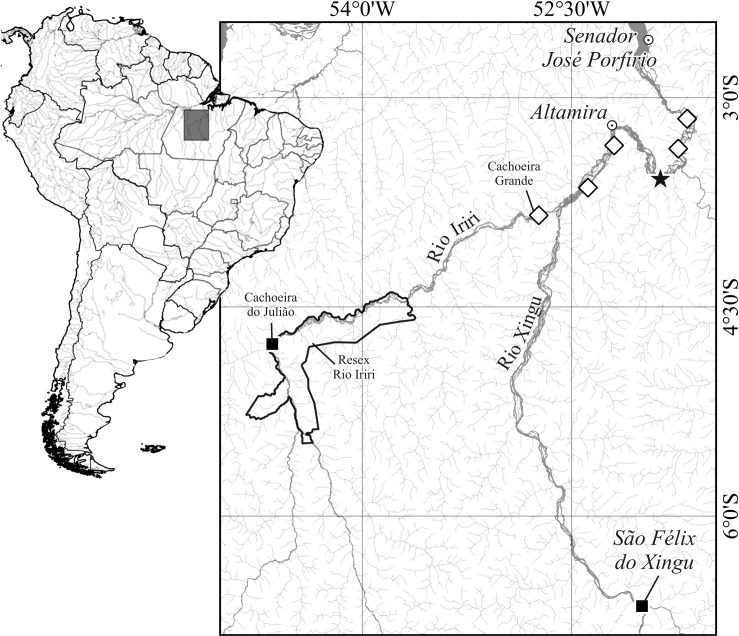
Geographic distribution of *Ossubtus xinguense* in Xingu River basin; (star) probable type locality in Volta Grande do Rio Xingu, (opened diamond) newly recorded sites on Iriri and Xingu rivers based on museum specimens, and (closed square) additional sites reported by local fishermen.

*Ossubtus xinguense* is endemic to the Xingu River basin, and confirmed from the rapids of Volta Grande do Xingu and the lower Rio Iriri, near its confluence with the Rio Xingu (see Material Examined). In addition, local fishermen report *O*. *xinguense* from the Rio Iriri Extractive Reserve (Resex do Rio Iriri at Cachoeira do Julião, 4°45'58"S 54°38'43"W), and from the Rio Xingu near the city São Félix do Xingu (Fig 14).

#### Ecological notes

**Fig 15:**
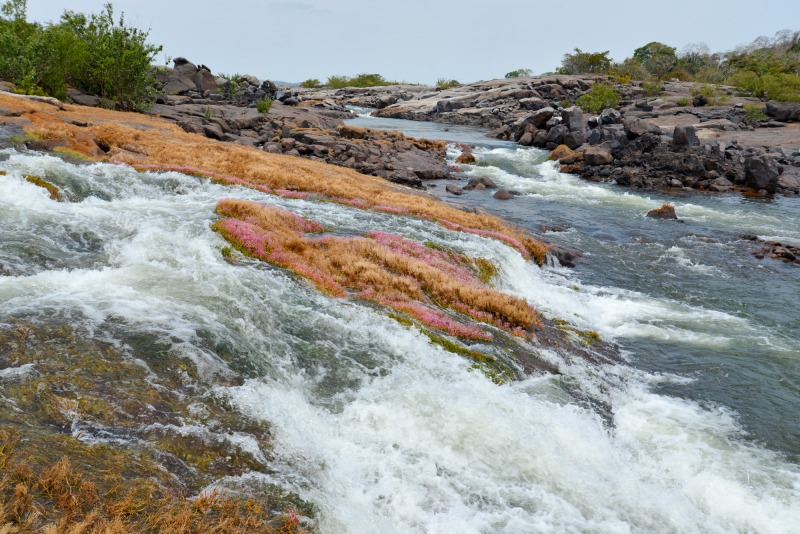
Rapids with rock outcrops covered by aquatic macrophytes of the family Podostemaceae. Cachoeira Grande of the Rio Iriri, main left bank tributary of Xingu River.

*Ossubtus xinguense* is caught in the rapids of the Xingu River along with other serrasalmids such as *Acnodon normani*, *Myloplus arnoldi*, *Myleus setiger* and *Tometes ancylorhynchus* and *Tometes kranponhah* [10], and many other rheophilic fishes of the families Characidae, Anostomidae and Loricariidae. To capture rheophilic species, fishermen normally throw cast nets in shallow areas of the rapids. Many rheophilic fishes shelter in crevices under rocks. After throwing the cast net, the fishermen plunges into the rapids to manually close the net and prevent fish from escaping. *Ossubtus xinguense* lives in clear swift waters over rocky outcrops covered by Podostemaceae, a habitat type that is common in the Middle Xingu River and lower Iriri (Fig 15). While snorkeling in rapids, we observed *O*. *xinguense* hiding under rocks over sandy bottoms.

Diet analysis of 10 adult specimens chosen randomly (GEA1729, 159.4–198.9 mm SL) revealed mainly Podostemaceae and Bryophyta (Frequency of Occurrence = 100% for each food item), allochthonous leaves (FO = 80%), twigs (FO = 50%), tree bark and plants roots (FO = 40% each), aquatic macroinvertebrates (FO = 20%), and sand grains (FO = 10%). Some adult specimens exhibit accumulations of lipids between bases of dorsal- and anal-fin membranes; those specimens were in reproductive condition. In all specimens, intestines were infested with an abundance of the nematode *Rondonia rondoni*. This nematode is possibly a symbiont rather than a parasite (Andrade *et al*., *in prep*.). Practically all fish are parasitized by metacercariae under the skin and scattered over body, head and fins, forming black spots known as “black-spot disease”. In addition, *O*. *xinguense* is parasitized by *Anphira xinguensis*, a gill isopod parasite exclusive to the species.

#### Morphometric analysis

**Fig 16:**
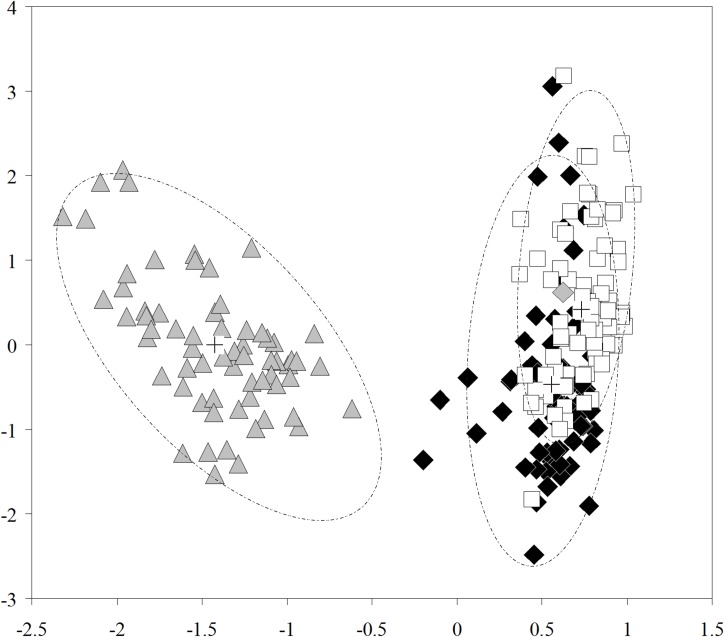
Scatter plot of scores on first (PC1, x-axis) and second (PC2, y-axis) principal components from morphometric analysis of *Ossubtus xinguense*. Juveniles (gray triangle) n = 68, Females (closed diamond) n = 65 including the holotype (gray diamond), Males (opened square) n = 83. Ellipses represent 95% confidence intervals, and crosses represent the centroid for each group.

Based on our PCA analyses, juveniles, and adult males and females of *O*. *xinguense* have similar overall body shapes. On the other hand, the analyses detected allometric differences between juveniles and adults, and males are generally larger than females. The PCA performed on 216 specimens established two distinct groups (Fig 16) represented by juveniles (N = 68, 36.4–92.1 mm SL) and adults (N = 148; 65 females 114.5–187.7 mm SL, and 83 males 150.9–228.6 mm SL). The two groups were clearly separated along PC1 (x-axis) with juveniles on the left and adults on the right (Fig 16), suggesting allometric differences during the growth. Linear measurements with significant differences between juveniles and adults (ANCOVA, F *=* 22.1, P << 0.01), included body depth and head length. The projection of individual scores on PC2 (y-axis), considered size-independent shape variation, shows full overlap between the two groups, suggesting juveniles and adults are similar in overall body shape. The measurements with the greatest loadings on PC2, such as 1st anal-fin lobe length, dorsal-fin length, postorbital distance, and fused 4th infraorbital width (Table 2), contribute to the highest variation. All other measurements are considered statistically indistinguishable with respect to growth (ANCOVA, d.f. = 214, n.s.), and therefore represent identical allometry and body shape between classes.

**Table 2:** Principal component scores calculated from 33 linear measurements (sexually dimorphic features excluded) taken on 216 specimens of *Ossubtus xinguense*. Loadings with absolute magnitude equal to or greater than 0.2 appear in bold.

	PC1	PC2
% Variance	98.406	0.269
Eigenvalue	1.770	0.005
SL (mm)	0.168	0.052
Body depth	0.176	-0.007
Head length	0.166	0.060
Supraoccipital process	0.175	0.026
Predorsal length	0.179	0.048
Dorsal-fin base length	0.179	0.025
Interdorsal length	0.195	-0.117
Adipose-fin base length	0.151	0.011
Caudal-peduncle depth	0.176	0.010
Anal-fin base length	0.179	0.027
Preanal length	0.161	0.041
Prepelvic length	0.152	0.072
Prepectoral length	0.139	0.126
Anal-pelvic distance	0.175	0.003
Pelvic-pectoral distance	0.164	0.008
Width of peduncle	0.179	0.037
Pectoral-fin length	0.166	0.012
Pelvic-fin length	0.171	-0.084
1st anal-fin lobe length	0.171	**-0.720**
Dorsal-fin length	0.172	**-0.422**
Distance from dorsal-fin origin to anal-fin origin	0.182	-0.024
Distance from dorsal-fin end to anal-fin origin	0.180	-0.032
Distance from dorsal-fin end to anal-fin end	0.177	0.005
Snout length	**0.210**	0.012
Interorbital width	0.191	0.019
Width head	0.165	-0.004
Postorbital distance	0.157	**0.217**
Fused 4th infraorbital width	0.150	**0.362**
Eye vertical diameter	0.143	-0.017
Mouth length	0.184	0.166
3rd infraorbital width	**0.243**	-0.030
Cheek gap width	0.144	0.184
Mouth width	0.187	0.065

Overall, the PCA summarizes morphological variation in *O*. *xinguense* (Table 2, Fig 16). The first principal component (PC1) accounts for 98.406% of total morphological variation, whereas PC2 accounts for 0.269%. PC1 correlates strongly with SL because the overall body size of the fish (represented by scores of the PC1) was significantly and positively correlated with the fish size (r = 0.987, P << 0.01). Therefore, the PC1 is interpreted as a general size factor and describes the allometric trajectory of body shape. The analysis of covariance performed between PC1 scores and log transformed SL of the species discloses that the two regressions of juveniles and adults have intercepts and slopes statistically different corroborating the separation between the two size classes (ANCOVA, F *=* 22.1, P << 0.01). Those results suggest strong allometric growth between juveniles and adults. PC2 is interpreted as a general descriptor of variation in body shape, free of allometry and only weakly correlated with SL (r = 0.082, n.s.). However, no differences were detected between both size classes or sexes with respect to overall shape (ANOVA, F = 2.08, n.s.). Thus, juveniles and adults, and males and females are indistinguishable with respect to overall shape excluding sexually dimorphic features.

## Discussion

*Ossubtus xinguense* is a strictly rheophilic fish that occurs in rapids associated with rocky substrates with crevices. Such habitats are especially difficult to sample, resulting in a scarcity of specimens in scientific collections and gaps in our fundamental understanding of the species. Based on an exhaustive analysis of newly collected material, it was possible to address questions on their taxonomic relationships, intraspecific morphological variation, distribution, and conservation status.

Morphology and Taxonomic relationships.‒ The most important characteristics for placing *O*. *xinguense* in the *Myleus* clade sensu Jégu [5] are the presence of incisiform teeth, two rows of premaxillary teeth with internal contact (no gap), the lack of spines in prepelvic serrature, and the wide olfactory fossa. For species of this clade, the incisiform teeth reflect a specialization for cutting soft leaves of Podostemaceae [3]. Jégu’s [5] report of caniniform teeth in the labial premaxillary row in *O*. *xinguense* is out of tune with other members of the *Myleus* clade. However, from analysis of several specimens of *O*. *xinguense*, including adult skeletons and cleared and stained juveniles, we notice that these teeth are actually incisiform with cutting edge poorly developed (Fig 1A). The caniniform shape in these teeth was refuted when we discovered in all specimens examined that the two anterior teeth of the labial premaxillary row are very anteroposteriorly flattened, with tooth base markedly developed laterally (Fig 1A). In adult specimens, each main cusp (most central) has scarcely developed lateral lobes, and is rounded to resemble a flattened spoon, like the remaining well-developed incisiform teeth. Therewith, all teeth are incisiform in *O*. *xinguense*, similar to the representative taxa of *Myleus* clade (i.e., *Myleus*, *Tometes* and *Mylesinus*). The two anterior labial teeth of *O*. *xinguense* were previously misunderstood as caniniform due to observations made on juvenile specimens.

The shift in mouth orientation in *O*. *xinguense*, hypothesized by Jégu [1], although not proved, was noticed here. Adults have a more inferiorly placed mouth than juveniles (Fig 5B, 45.6 mm SL). This subtle, difference results from a ventrally directed bend in the neurocranium during growth. Although the rotation of neurocranium is not statistically supported by snout length (ANCOVA, n.s.), the higher score value at PC1 for this measure (see Table 1) indicates greater differences between juveniles and adults specimens than other linear measures. In addition, *O*. *xinguense* has a shallow, elongated neurocranium (Fig 6), whereas members of the *Myleus* clade have a relatively deep, triangular neurocranium. The elongate neurocranium in *O*. *xinguense* is similar to the condition found in *Colossoma* and *Piaractus*. However, the neurocranium of *O*. *xinguense* is slender and light (like the neurocranium of *Myleus*, *Tometes* and *Mylesinus*) compared to the robust neurocranium of *Colossoma* and *Piaractus* (both seed-crushing genera). This condition is exemplified by the wide aperture of the olfactory fossa in *O*. *xinguense*, resulting from the thin mesethmoid roof. *Colossoma* and *Piaractus* have olfactory fossa with narrow aperture and stout mesethmoid roof. The wide olfactory fossa is exclusive to the *Myleus* clade within Serrasalmidae [5], and presumably houses a large sensory organ used to find food and perhaps mates. According to Jégu [5]: 323 (character 16), *Ossubtus* also shares with *Colossoma* and *Piaractus* a reduced number of branched anal-fin rays (i.e., *Ossubtus* with 22–25 and *Colossoma-Piaractus* with 20–24 versus 26–34 in other members of the *Myleus* clade sensu Jégu [5]). That reduction is considered a reversal in *Ossubtus*.

According to Jégu [5]: 365 (character 112) the presence of four infraorbitals in *Ossubtus* is an autapomorphy and results from the fusion of infraorbitals 3 and 4. Most species of Serrasalmidae have six or sometimes five infraorbitals (e.g., members of the piranha clade according to Machado-Allison [14]). Curiously, the reduced number of infraorbitals in *Ossubtus* is shared with some species of *Leporinus* (Anostomidae) that similarly have a subinferior to inferior mouth [49]. Thereby, the fusion of infraorbitals might be related to the downward re-orientation of neurocranium or by modifications (e.g., elongation [49]) in some bones of neurocranium in taxa with ventrally directed mouths.

Sexual dimorphism and Allometric variation.*‒ Ossubtus xinguense* shares with herbivorous Serrasalmidae, such as the species of the genera *Acnodon*, *Metynnis*, *Myleus*, *Mylesinus*, *Tometes*, *Utiaritichthys* [3], and *Myloplus* [35] dimorphic features mainly evidenced by the anal fin wherein mature males have an additional lobe formed by elongation of the middle rays which sometimes bear divergent hooks distally (Fig 12), and dorsal fin with long and thin filaments. However, those sexually dimorphic features in *O*. *xinguense* also seem correlated to variation in physiological condition. Among males greater than 180 mm SL, 70% showed the stiff divergent hooks on anal-fin rays (Fig 12), and less of 10% of males larger than 160 mm SL showed filaments on dorsal-fin rays. The dorsal-fin extensions in *O*. *xinguense* are noticeably smaller than those found in the aforementioned genera. Some authors (e.g. [50]) express doubts on the permanence of sexually dimorphic features in serrasalmids, and consider them to be restricted to the breeding season as in other characiforms (e.g. [51], [52]). However, it is known that in Serrasalmidae the dimorphic features do not disappear once established, and remain evident in mature males outside of the breeding period.

We expected to find morphometric differences in body shape between males and females. That hypothesis was refuted by the multivariate analysis performed in this study. However, the allometric distinction between juveniles and adults [1] was supported in *O*. *xinguense*. The results may indicate ecomorphological trends associated with rheophilic behavior of *O*. *xinguense* that, like other reophilic serrasalmids, show up to 100 mm SL allometric differences between juveniles and adults (see Figure 2a in [7], and Figure 3a in [8] and [33]). Therefore, allometric bodily differences are possibly associated with skill of juveniles remain in the rapids without being carried away by the current.

Conservation.‒ This study establishes the downstream limit of *O*. *xinguense* as the last major rapids of the Xingu River (Fig 14), specifically Cachoeiras Tapaiúna and Itamaracá. The farthest upstream record confirmed for *O*. *xinguense* is the last large cachoeira on its left bank tributary, the Rio Iriri. Local fishermen claim that the species occurs in rapids further upstream, such as Cachoeira do Julião located in Rio Iriri Extractive Reserve (Fig 14). Although infrequently, *O*. *xinguense* is sold for human consumption at street markets in São Félix do Xingu (Fig 14), suggesting it occurs further upstream in the Xingu River as well. Based on those reports, we believe that *O*. *xinguense* is widely yet irregularly distributed in the rapids of the Middle Xingu River.

The previous scarcity of *O*. *xinguense* in fish collections suggested that the species is rare in nature (see Jégu and Zuanon [2]). However, their rarity in museums is better explained by a limited ability to effectively sample rapids. Based on underwater observations, *O*. *xinguense* commonly inhabits rocky crevices covered by aquatic macrophytes of the family Podostemaceae (Fig 15). Unlike other rheophilic serrasalmids, juveniles and adults of *O*. *xinguense* seems restricted to sheltered portions of rapids areas and seldom move between rocky outcrops with groves of Podostemaceae. Other serrasalmids (e.g. *Myleus setiger*, *Myloplus rhomboidalis*, *Mylesinus* spp., and *Tometes* spp.) are often found in sheltered portions of rapids, as well as in open swift current and calmer stretches upstream and downstream of rapids. Our observations support a stronger affinity in *O*. *xinguense* for habitats closely associated with swift rapids and ample groves of Podostemaceae. The degradation of the rapids and their conversion to reservoirs by hydroelectric dams is the main cause of loss of rheophilic diversity. Zuanon and Jégu [38] classified *O*. *xinguense* as ‘Endangered’ according to the World Conservation Union. Despite the expansion of the known range of *O*. *xinguense*, serious threats to the species remain: the Belo Monte Dam Complex. One dam, Pimental, will flood approximately 80 river kms of the Xingu channel; approximately 90 river kms from below the Pimental dam to above the outflow of a second dam, Belo Monte, will be dewatered [53]. The flood pulse of the dewatered stretch will be severely attenuated and likely impact stands of Podostemaceae. The irregular distribution of *O*. *xinguense* and its endemism to the Xingu Basin reinforce the species’ original threat category. Therefore, we recommend an increase of studies to establish conservation units for proper management. Furthermore, the conservation of rapids upstream of the Belo Monte impact area is mandatory to assure the health of the remaining populations of rheophilic fishes in the Xingu River.
